# Supplementary material for: NE contribution to rebooting unconsciousness caused by midazolam
Source: eLife. 2024 Nov 20;13:RP97954. doi: 10.7554/eLife.97954 (PMC11578583; doi:10.7554/eLife.97954)
Supplement: Supplementary file 1. [file elife-97954-supp1.docx]

**Supplemental information**

**NE contribution to rebooting unconsciousness caused by midazolam**

LeYuan Gu^1,2#^, WeiHui Shao^2#^, Lu Liu^1#^, Qing Xu^1#^, YuLing Wang^2#^, JiaXuan Gu^2#^, Yue Yang^2^, ZhuoYue Zhang^1^, YaXuan Wu^2^, Yue Shen^3^, Qian Yu^2^, XiTing Lian^1^, Haixiang Ma^5^, YuanLi Zhang^2^, HongHai Zhang^1,2,3,4^*

^1^Department of Anesthesiology, Zhejiang University School of Medicine, Hangzhou, 310006, China

^2^Department of Anesthesiology, the Fourth Clinical School of Medicine, Zhejiang Chinese Medical University, Hangzhou, 310006, China

^3^Department of Anesthesiology, Affiliated Hangzhou First People's Hospital, Westlake University School of Medicine, Hangzhou, 310006, China

^4^Westlake Laboratory of Life Sciences and Biomedicine, Hangzhou, 310006, China

^5^Medical College of Jining Medical University, Ningji, 272067, Shandong, China

^#^These authors contributed equally to this work

Corresponding authors: HongHai Zhang

Email address: [zhanghonghai_0902@163.com](mailto:zhanghonghai_0902@163.com) & zhh0902@zju.edu.cn

ORCID: http://orcid.org/0000-0003-3530-2060

**Supplemental Table 1. Summary of experimental groups of C57B/6J mice**

| **Figure** | **Experimental groups** | **Numbers of mice** |
| --- | --- | --- |
| **Figure 1** | Midazolam (40mg/kg) | 7 |
|  | Midazolam (50mg/kg) | 8 |
|  | Midazolam (60mg/kg) | 8 |
|  | Midazolam (70mg/kg) | 8 |
|  | ELISA-Vehicle | 6 |
|  | ELISA-Midazolam (60mg/kg) | 6 |
|  | Vehicle | 8 |
|  | Atomoxetine (10mg/kg) | 8 |
|  | Atomoxetine (20mg/kg) | 8 |
|  | Vehicle | 6 |
|  | DSP-4 (3 days) | 6 |
|  | DSP-4 (10 days) | 6 |
|  | Vehicle+Vehicle | 6 |
|  | Vehicle+Atomoxetine | 6 |
|  | DSP-4 (3 days)+Atomoxetine | 6 |
|  | DSP-4 (10 days)+Atomoxetine | 6 |
| **Figure 2** | Midazolam (60mg/kg) | 6 |
|  | Vehicle | 6 |
|  | DSP-4 (10 days) | 6 |
|  | Vehicle+Vehicle | 6 |
|  | Vehicle+Atomoxetine | 6 |
|  | DSP-4 (10 days)+Atomoxetine | 6 |
| **Figure 3** | No light | 8 |
|  | 20min 2mW Photostimulation (PS) | 8 |
|  | 20min 4mW PS | 8 |
|  | No light | 7 |
|  | 10min 4mW PS | 7 |
|  | 20min 4mW PS | 7 |
|  | No PS | 7 |
|  | Left PS | 7 |
|  | Right PS | 7 |
|  | Bilateral PS | 7 |
|  | No PS | 6 |
|  | PS | 6 |
|  | Vehicle | 6 (male) |
|  | CNO (0.1mg/kg) | 6 (male) |
|  | CNO (0.2mg/kg) | 6 (male) |
|  | Vehicle | 6 (female) |
|  | CNO (0.1mg/kg) | 6 (female) |
|  | CNO (0.2mg/kg) | 6 (female) |
|  | Vehicle | 6 (male) |
|  | Left Chemical stimulation (CS) | 6 (male) |
|  | Vehicle | 6 (female) |
|  | Left Chemical stimulation (CS) | 6 (female) |
|  | Vehicle | 6 (male) |
|  | Right CS | 6 (male) |
|  | Vehicle | 6 (female) |
|  | Right CS | 6 (female) |
|  | Vehicle | 6 (male) |
|  | Bilateral CS | 6 (male) |
|  | Vehicle | 6 (female) |
|  | Bilateral CS | 6 (female) |
|  | No CS | 6 (male) |
|  | CS | 6 (male) |
|  | No CS | 6 (female) |
|  | CS | 6 (female) |
| **Figure 4** | No PS | 6 |
|  | PS | 6 |
|  | No CNO | 6 (male) |
|  | CNO | 6 (male) |
|  | No CNO | 6 (female) |
|  | CNO | 6 (female) |
| **Figure 5** | No PS | 8 |
|  | PS | 8 |
|  | Vehicle | 8 (male) |
|  | CNO | 8 (male) |
|  | Vehicle | 8 (female) |
|  | CNO | 8 (female) |
| **Figure 6** | Vehicle | 8 |
|  | Phenylephrine (10mg/ml) | 8 |
|  | Phenylephrine (20mg/ml) | 8 |
|  | Vehicle | 8 |
|  | Prazosin (0.75mg/ml) | 8 |
|  | Prazosin (1.5mg/ml) | 8 |
|  | Vehicle | 9 |
|  | Clonidine (0.75mg/ml) | 9 |
|  | Clonidine (1.5mg/ml) | 9 |
|  | Vehicle | 8 |
|  | Yohimbine (15umol/ml) | 8 |
|  | Yohimbine (22.5umol/ml) | 8 |
|  | Yohimbine (30umol/ml) | 8 |
|  | Vehicle | 7 |
|  | Isoprenaline (2mg/ml) | 7 |
|  | Isoprenaline (4mg/ml) | 7 |
|  | Vehicle | 6 |
|  | Propranolol (2.5mg/ml) | 6 |
|  | Propranolol (5mg/ml) | 6 |
|  | Vehicle+Vehicle | 6 |
|  | Vehicle+Phenylephrine (20mg/ml) | 6 |
|  | Vehicle+Propranolol (5mg/ml) | 6 |
|  | Phenylephrine (20mg/ml)+Propranolol (5mg/ml) | 6 |
|  | No PS+Vehicle (ICV) | 7 |
|  | PS+Vehicle (ICV) | 7 |
|  | PS+Prazosin (ICV) | 7 |
|  | No PS+Prazosin (ICV) | 7 |
|  | No PS+Vehicle (VLPO) | 6 |
|  | PS+Vehicle (VLPO) | 6 |
|  | PS+Prazosin (VLPO) | 6 |
|  | No PS+Prazosin (VLPO) | 6 |
|  | Vehicle+Vehicle (ICV) | 6 |
|  | CNO+Vehicle (ICV) | 6 |
|  | CNO+Prazosin (ICV) | 6 |
|  | Vehicle+Prazosin (ICV) | 6 |
|  | Vehicle+Vehicle (VLPO) | 7 (male) |
|  | CNO+Vehicle (VLPO) | 7 (male) |
|  | CNO+Prazosin (VLPO) | 7 (male) |
|  | Vehicle+Prazosin (VLPO) | 7 (male) |
|  | Vehicle+Vehicle (VLPO) | 7 (female) |
|  | CNO+Vehicle (VLPO) | 7 (female) |
|  | CNO+Prazosin (VLPO) | 7 (female) |
|  | Vehicle+Prazosin (VLPO) | 7 (female) |
| **Figure 7** | Vehicle+No PS | 6 |
|  | Vehicle+PS | 6 |
|  | Prazosin+PS | 6 |
|  | Prazosin+No PS | 6 |
|  | Vehicle+No CNO | 6 |
|  | Vehicle+CNO | 6 |
|  | Prazosin+CNO | 6 |
|  | Prazosin+No CNO | 6 |
| **Figure 8** | Vehicle (ICV) | 7 |
|  | Gabazine (2ug/ml) (ICV) | 7 |
|  | Gabazine (4ug/ml) (ICV) | 7 |
|  | Vehicle (LC) | 6 |
|  | Gabazine (2ug/ml) (LC) | 6 |
|  | Gabazine (4ug/ml) (LC) | 6 |
|  | Vehicle+Vehicle | 6 |
|  | Gabazine+Vehicle | 6 |
|  | Vehicle+Prazosin | 6 |
|  | Gabazine+Prazosin | 6 |
| **Figure 9** | Sham | 6 |
|  | shRNA | 6 |
|  | Midazolam (40mg/kg) | 8 |
|  | Midazolam (50mg/kg) | 8 |
|  | Midazolam (60mg/kg) | 8 |
|  | Midazolam (70mg/kg) | 8 |
|  | Sham | 8 |
|  | shRNA | 8 |
|  | Sham | 6 |
|  | shRNA | 6 |
|  | Sham | 8 |
|  | shRNA+Vehicle | 8 |
|  | shRNA+Prazosin | 8 |

**Supplemental Table 2. Summary of the total number of C57B6J mice**

| **Figure** | **Numbers of mice** |
| --- | --- |
| **Figure 1** | **109 (male)** |
| **Figure 2** | **36 (male)** |
| **Figure 3** | **217 (151 male+66 female)** |
| **Figure 4** | **36 (24 male+12 female)** |
| **Figure 5** | **48 (44 male+16 female)** |
| **Figure 6** | **302 (274 male+28 female)** |
| **Figure 7** | **48 (male)** |
| **Figure 8** | **63 (male)** |
| **Figure 9** | **96 (male)** |
| **Total number of mice** | **955 (832 male+122 female)** |

**Supplemental Table3. Statistical analysis**

| **Figure** | **Comparisons** | **Test** |
| --- | --- | --- |
| **Figure 1** | Content of NE | Two-way ANOVA |
|  | Recovery time | Ordinary one-way ANOVA |
|  | Normalized TH^+^ cell number | Unpaired t-test |
|  | Quantification of c-fos(+)/TH(+) cells | Unpaired t-test |
| **Figure 2** | △F/F peak | Unpaired t-test |
|  | Quantification of c-fos(+)/TH(+) cells | Unpaired t-test |
|  | Recovery time | Ordinary one-way ANOVA |
|  | Normalized TH^+^ cell number | Unpaired t-test |
| **Figure 3** | Recovery time | Ordinary one-way ANOVA |
|  | Quantification of c-fos(+)/TH(+) cells | Unpaired t-test |
| **Figure 4** | Recovery time | Ordinary one-way ANOVA |
|  | △F/F peak | Unpaired t-test |
| **Figure 5** | Recovery time | Ordinary one-way ANOVA |
| **Figure 6** | Recovery time | Ordinary one-way ANOVA |
| **Figure 7** | Delta wave | Two-way ANOVA |
|  | Theta wave | Two-way ANOVA |
|  | Alpha wave | Two-way ANOVA |
|  | Beta wave | Two-way ANOVA |
|  | Gamma wave | Two-way ANOVA |
| **Figure 8** | Recovery time | Ordinary one-way ANOVA |
|  | △F/F peak | Unpaired t-test |
| **Figure 9** | Quantification of  GABAA-R(+)/TH(+) cells | Unpaired t-test |
|  | Recovery time | Ordinary one-way ANOVA |
|  | △F/F peak | Unpaired t-test |

**Supplemental Table 4. Reagent or resource**

| **Reagent or resource** | **Source** | **Identifier** |
| --- | --- | --- |
| **Antibodies** |  |  |
| Rabbit anti-c-fos | Cell Signaling Technology | 2250S |
| Mouse anti-TH | Merck-Millipore | MAB318 |
| Rabbit anti-TH | Merck-Millipore | MAB152 |
| Mouse anti-GABAA-R | Abcam | ab94585 |
| Donkey anti-mouse Alexa 546 | Thermo Fisher Scientific | A10036 |
| Donkey anti-mouse Alexa 488 | Thermo Fisher Scientific | A21202 |
| Goat anti-rabbit Cy5 | Thermo Fisher Scientific | A10523 |
| Donkey anti-rabbit Alexa 488 | Thermo Fisher Scientific | A21206 |
| Donkey anti-rabbit Alexa 546 | Thermo Fisher Scientific | A10040 |
| **Bacterial and virus strains** |  |  |
| rAAV-Dbh-GCaMP6m-WPRE-hGH pA | Brain VTA Technology Co., Ltd. | N/A |
| rAAV-Ef1α-DIO-hM3D(Gq)-mCherry | Brain VTA Technology Co., Ltd. | N/A |
| rAAV-Ef1α-DIO-hChR2(H134R)-EYFP | Brain VTA Technology Co., Ltd. | N/A |
| rAAV-mTh-NLS-CRE-WPRE-SV40 polyA | Brain VTA Technology Co., Ltd. | N/A |
| rAAV-Dbh-EGFP-S'miR-30a-shRNA(GABAA receptor)-3’-miR30a-WPREs | Brain VTA Technology Co., Ltd. | N/A |
| rAAV-Dbh-CRE-WPRE-hGH pA | Brain VTA Technology Co., Ltd. | N/A |
| **Chemicals, peptides, and recombinant proteins** | |  |
| Atomoxetine | Sigma-Aldrich | Ca#Y0001586 |
| DSP-4 | Sigma-Aldrich | C8417 |
| Enzyme-linked immunosorbent assay kit | Yan Sheng Biological Technology Co., Ltd | YS-M195 |
| Midazolam | Jiangsu Nhwa Pharmaceutical Co., Ltd | N/A |
| Gabazine | MedChemExpress | HY-103533 |
| Phenylephrine | MedChemExpress | HY-B0471 |
| Prazosin | MedChemExpress | HY-B0193 |
| Clonidine | MedChemExpress | HY-B0409 |
| Yohimbine | Aladdin | Y111137 |
| Isoprenaline | Sigma-Aldrich | [I5627](https://www.sigmaaldrich.cn/CN/zh/product/sigma/i5627) |
| Propranolol | MedChemExpress | HY-B0573 |
| Clozapine N-oxide (CNO) | MedChemExpress | HY-17366 |
| **Experimental models: Organisms/strains** | | |
| C57BL/6J mice | the Animal Center of Zhejiang University School of Medicine | N/A |
| **Software and algorithms** |  |  |
| ImageJ | NIH, Bethesda, MD, USA | https://imagej.nih.gov/ij/ |

**Supplemental Videos**

| **Videos** | **Content** | **Related Figure** |
| --- | --- | --- |
| **Optogenetic experiments** | | **Figure 3** |
| **Video 1** | An example of a C57BL/6J mouse intervened with midazolam, and without photostimulation to LC, demonstrating the long recovery time. |  |
| **Video 2** | An example of a C57BL/6J mouse intervened with midazolam, and with photostimulation to LC, demonstrating the shorter recovery time. |  |
| **Chemogenetic experiments** | |  |
| **Video 3** | An example of a male C57BL/6J mouse intervened with midazolam, and without the chemogenetic activation of the LC^NE^ neurons, demonstrating the long recovery time. |  |
| **Video 4** | An example of a male C57BL/6J mouse intervened with midazolam, and with the chemogenetic activation of the LC^NE^ neurons, demonstrating the shorter recovery time |  |
| **Video 5** | An example of a female C57BL/6J mouse intervened with midazolam, and without the chemogenetic activation of the LC^NE^ neurons, demonstrating the long recovery time. |  |
| **Video 6** | An example of a female C57BL/6J mouse intervened with midazolam, and with the chemogenetic activation of the LC^NE^ neurons, demonstrating the shorter recovery time. |  |
| **EEG experiments** | | **Figure 7** |
| **Video 7** | An example of a C57BL/6J mouse intervened with midazolam, without photostimulation to LC, and with ICV injection of the vehicle, recording EEG, demonstrating the long recovery time. |  |
| **Video 8** | An example of a C57BL/6J mouse intervened with midazolam, with photostimulation to LC, and with ICV injection of the vehicle with EEG recordings to reverse the effects of photostimulation, demonstrating the shorter recovery time. |  |
| **Video 9** | An example of a C57BL/6J mouse intervened with midazolam, with photostimulation to LC with ICV injection of prazosin with EEG recordings to reverse the effects of photostimulation, demonstrating the shorter recovery time. |  |
| **Video 10** | An example of a C57BL/6J mouse intervened with midazolam, without the chemogenetic activation of the LC^NE^ neurons with ICV injection of the vehicle, recording EEG, demonstrating the long recovery time. |  |
| **Video 11** | An example of a C57BL/6J mouse intervened with midazolam, with the chemogenetic activation of the LC^NE^ neurons with ICV injection of the vehicle, recording EEG, demonstrating the shorter recovery time. |  |
| **Video 12** | An example of a C57BL/6J mouse intervened with midazolam, with the chemogenetic activation of the LC^NE^ neurons with ICV injection of prazosin with EEG recordings to reverse the effects of chemogenetic activation, demonstrating the long recovery time. |  |
| **LC^NE^ neurons GABAA-R knockdown experiments** | | **Figure 9** |
| **Video 13** | An example of a C57BL/6J mouse intervened with midazolam, and without knocking down GABAA-R on the LC^NE^ neurons. |  |
| **Video 14** | An example of a C57BL/6J mouse intervened with midazolam, and with knocking down GABAA-R on the LC^NE^ neurons, demonstrating the shorter recovery time. |  |
| **Video 15** | An example of a C57BL/6J mouse intervened with midazolam, and with knocking down GABAA-R on the LC^NE^ neurons without VLPO microinjection of the prazosin, demonstrating the shorter recovery time. |  |
| **Video 16** | An example of a C57BL/6J mouse intervened with midazolam, and with knocking down GABAA-R on the LC^NE^ neurons with VLPO microinjection of the prazosin, demonstrating the long recovery time. |  |
